# Where is iron in erionite? A multidisciplinary study on fibrous erionite-Na from Jersey (Nevada, USA)

**DOI:** 10.1038/srep37981

**Published:** 2016-11-28

**Authors:** Alessandro F. Gualtieri, Nicola Bursi Gandolfi, Simone Pollastri, Kilian Pollok, Falko Langenhorst

**Affiliations:** 1 Chemistry and Earth Sciences Department, The University of Modena and Reggio Emilia, Via Campi 103, I-41125 Modena (Italy); 2 Institut für Geowissenschaften Mineralogie, Friedrich-Schiller-Universität Jena, Carl-Zeiss-Promenade 10, D-07745 Jena (Germany)

## Abstract

Fibrous erionite is a mineral fibre of great concern but to date mechanisms by which it induces cyto- and geno-toxic damage, and especially the role of iron associated to this zeolite species, remain poorly understood. One of the reasons is that we still don’t know exactly where iron is in natural erionite. This work is focused on fibrous erionite-Na from Jersey (Nevada, USA) and attempts to draw a general model of occurrence of iron in erionite and relationship with toxicity mechanisms. It was found that iron is present as 6-fold coordinated Fe^3+^ not part of the zeolite structure. The heterogeneous nature of the sample was revealed as receptacle of different iron-bearing impurities (amorphous iron-rich nanoparticles, micro-particles of iron oxides/hydroxides, and flakes of nontronite). If iron is not part of the structure, its role should be considered irrelevant for erionite toxicity, and other factors like biopersistence should be invoked. An alternative perspective to the proposed model is that iron rich nano-particles and nontronite dissolve in the intracellular acidic environment, leaving a residue of iron atoms at specific surface sites anchored to the windows of the zeolite channels. These sites may be active later as low nuclearity groups.

Erionite is a natural zeolite that may occur as diagenetic product in sedimentary environment^1^, as product of hydrothermal alteration^2^, or in the vugs of altered basalts^3^. It belongs to the so-called ABC-6 family of zeolites^4^, whose members are originating from the stacking along the *c*-axis of layers of six-membered rings made of (Si,Al)O_4_ tetrahedra, following an ABC scheme. Erionite is characterized by a 6-layer repetition, with hexagonal symmetry, space group *P*6_3_/*mmc*, unit cell *a* ≅ 1.315 nm, *c* ≅ 1.505 nm, and ideal formula K_2_(Na, Ca_0.5_)_7_[Al_9_Si_27_O_72_]·28H_2_O^5^. A large chemical variability characterizes this zeolite, with three different species identified according to the most abundant extra-framework cation: erionite-Na, erionite-K, and erionite-Ca^6^,^7^. The framework of erionite contains two double-6 rings (D6R), two cancrinite (ε) cages, and two erionite (23-hedron) cages per unit cell. Cancrinite and erionite cages host the extraframework cations (EF). The cancrinite cage contains a K^+^ ion located at the centre of the cavity, whereas several EF cation sites are placed along the *c* axis of the erionite cavity^8^,^9^,^10^,^11^. H_2_O molecules are arranged around the axis of the erionite cavity providing coordination to the EF cations.

Natural fibrous erionite has recently gained great concern because its environmental exposure has been linked to the outbreak of malignant mesothelioma (MM) epidemics in several villages of Central Anatolia, Turkey^12^,^13^. *In vivo* studies have unambiguously proven that fibrous erionite is by far more carcinogenic than chrysotile and crocidolite^14^, and for this reason, it has been included by the International Agency for Research on Cancer (IARC) in the Group 1 Human-Carcinogen list (IARC 1987; 2011). Initially, environmental exposure to erionite has been hastily considered as locally circumscribed to Central Anatolia but in 1981, the first North American case of erionite-related lung disease was identified in Utah (USA)^15^ and several others were subsequently reported^16^. Nowadays, the concern with the carcinogenic potential of erionite is of public interest in the USA and Europe and recent biological activity of erionite samples from different localities has been demonstrated to be similar^17^. Although the toxic potential of erionite is out of discussion, the mechanisms by which it induces cyto- and geno-toxic damage remain poorly understood. One of the reasons is the great chemical variability and molecular arrangement of natural fibrous erionites. A key factor contributing to its toxicity potential is deemed to be the presence of iron^18^,^19^,^20^. Iron in mineral fibres may be responsible for carcinogenic activity namely via ROS/RNS production during the phagocytosis induced inflammatory burst^21^. Specifically, active iron present at the surface of the fibres promotes the formation of reactive HO^•^ species by the surface Fenton reaction chain. Reactive HO^•^ are responsible for secondary genotoxicity via damage to proteins and DNA, cell injury/mutation, nucleotide coenzyme destruction, membrane damage, apoptosis, lipid peroxidation^22^, and fibre encapsulation by collagen and iron-rich proteins^23^. Hence, the knowledge of the crystal chemistry of iron-bearing fibrous erionite is of absolute importance for understanding the mechanisms that prompt its bio-toxic action. But where is iron in erionite? The answer to this question is a hot topic, open to debate, with apparently disconnected, inconclusive and contradictory evidences reported in the specific literature. Theoretically, the following chemical environments are possible for iron in erionite: (1) Fe^2+^, and to a minor extent Fe^3+^, may virtually replace for Si^4+^ and Al^3+^ in the zeolite framework^24^; (2) Fe^3+^ and Fe^2+^ can be found in the extraframework cavities (micropores) as octahedral Fe(H_2_O)_6_ clusters; (3) Fe^3+^, and to a very minor extent Fe^2+^, can be associated with iron-rich impurities (both crystalline and amorphous oxides, hydroxides or sulphates) present as particles or nanoparticles coating the surface of the fibres.

Regarding model (1), the analysis of erionite-K from the Cappadocia region of Turkey obtained from TEM-EDS analysis published by Dogan^25^ K_3.09_Na_0.26_Ca_1.57_Mg_0.55_[Al_6.61_Si_28.70_Fe^3+^_0.60_O_72_] apparently point to Fe^3+^ hosted in the zeolite framework but no justification to that assignment is given. The EPMA analysis of several fibrous erionite-K (and offretite) samples from the Killdeer Mountains, Dunn County (North Dakota, USA) displays an extremely variable iron content (003–1.99 wt% Fe_2_O_3_) that was also arbitrarily assigned as Fe^3+^ hosted in the zeolite framework^26^.

Regarding model (2), natural samples with iron hosted in the erionite micropores have never been reported to date. Oppositely, Fe^2+^-exchanged erionites have been obtained in laboratory^27^,^28^. According to Ballirano *et al*.^28^, Fe^2+^ is fixed in the erionite cage and is six-fold coordinated to H_2_O molecules. The occurrence of Fe^2+^ within the erionite cage causes a gradual migration of the other EF cations and in addition, induces a small rearrangement of the H_2_O molecules.

As far as model (3) is concerned, in erionite-K from Rome (Oregon, USA), 95% of iron was attributed to Fe^3+^-bearing, super-paramagnetic, oxide-like nanoparticles with dimensions between 1 and 9 nm, and the remaining 5% was attributed to hematite particles with size ≥10 nm, both located at the crystal surface^10^. Later, Matassa *et al*.^29^ discovered that the same erionite-K from Rome actually contains aggregates of nontronite, an iron-bearing smectite of ideal formula (Ca_0.5_, Na)_0.33_Fe^3+^_2_(Si_3.67_Al_0.33_)O_10_(OH)_2_·nH_2_O. Successive EDX analysis of the fibres leached with simulated lung fluids (SLF) indicated the absence of iron due to the removal of nontronite from their surface produced by the leaching process, confirming the absence of iron in the structure of erionite. The same sample has been selected for a BSE-EDS analysis combined with micro-Raman spectroscopy and iron has been identified as component of iron-bearing microcrystals of hematite (α-Fe_2_O_3_), goethite (α-FeOOH), and jarosite KFe_3_(SO_4_)_2_(OH)_6_ nucleated at the surface of the fibres^30^. Iron was not hosted in the crystal structure, although the detection limits of the applied methods do not permit to unequivocally rule out this possibility. The same authors found that iron in fibrous erionite-K from Karlik (Cappadocia, Turkey) was as well present as iron-bearing sub-micrometric crystals of hematite and goethite coating the surface of the fibres^30^, and not in the crystal structure.

Minor amounts of iron (Fe_2_O_3_ < 0.13 wt%) were determined but not interpreted for fibrous erionite-K from Lander County (Nevada, USA) and Karain (Turkey)^31^. Iron has also been qualitatively detected but not interpreted in a fibrous (presumably) erionite-Na found in the lung tissue of a North American patient who was raised on a farm in the Mexican Volcanic Belt region and died of epithelial pleural MM^32^.

This work is focused on the full characterization of a representative sample of fibrous erionite-Na from Jersey (Nevada, USA) which contains iron from the micro- to the atomic scale. The detailed study of the crystal structure, with special attention to iron is an attempt to put the pieces of the puzzle together, draw a general model of occurrence of iron in erionite and revise its toxicity model in respect to iron chemistry. Preliminary information on the chemical environment of iron in the sample has been attained from Mössbauer and X-ray absorption spectroscopy experiments^33^.

## Results

Table 1 reports the interatomic distances of fibrous erionite-Na from Jersey calculated from the Rietveld refinement using the synchrotron data at the absorption K-edge of iron. Identical results were obtained using the data away from the absorption edge. The major result is that no evidence was found to support the presence of iron in both the framework or extraframework cavities, despite several attempts using different refinement strategies and chemistry constraints (site population constrained to the iron content from the chemical analysis).

**Figure 1:**
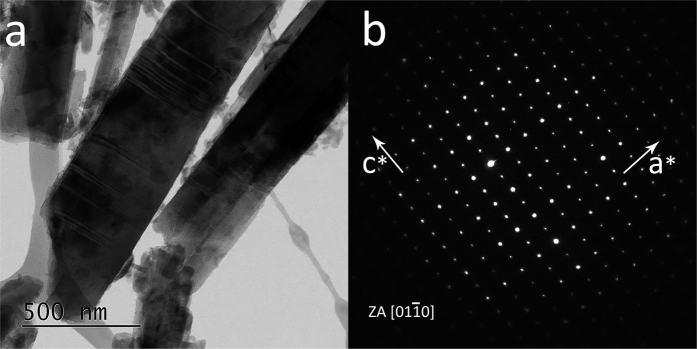
High resolution TEM image of fibrous erionite-Na. TEM imaging of fibres of erionite-Na showing signs of stacking disorder along c (**a**) and the relative SAED pattern from an area of high cristallinity with zone axis [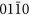] (**b**). Diffraction spots 000 *l* with *l* = 2n + 1 in the [000 *l*]* rows apparently break the conditions imposed by the systematic absences for that space group *P*6_3_/*mmc*, but this is a physical effect due to multiple diffraction effects from a thick section of the specimen under the beam.

**Figure 2:**
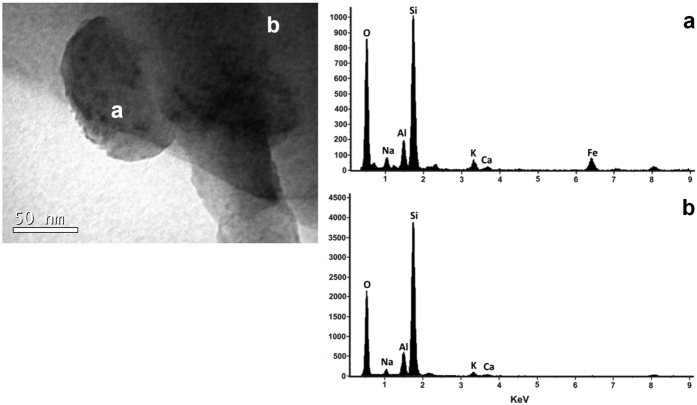
Nanoparticles at the surface of the erionite-Na fibres. High-resolution TEM image of erionite sample with spherical iron-bearing nanoparticles at the surface of the fibres. The EDS spectra evidence that the nanoparticles are enriched in iron with respect to the fibre bulk.

**Figure 3:**
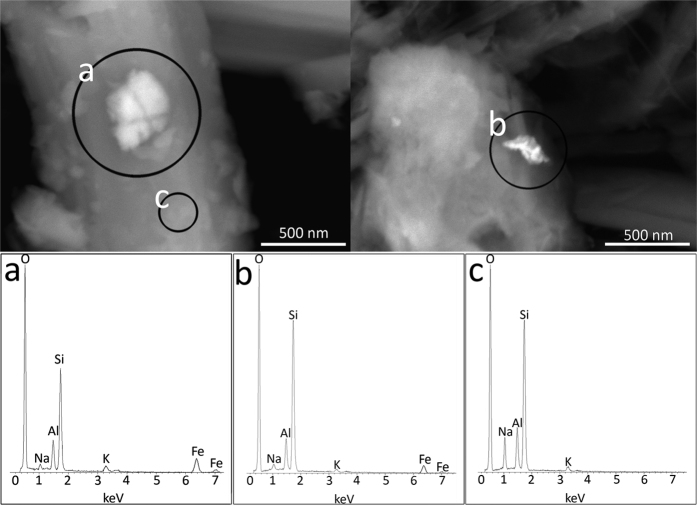
FEG/SEM images and EDS of the erionite-Na fibres. Submicronic clusters of iron-rich particles at the surface of the erionite-Na fibres captured during a FEG/SEM session and relative EDS witnessing the enrichment in iron (**a** and **b**) with respect to the bulk of the fibre (**c**).

**Figure 4:**
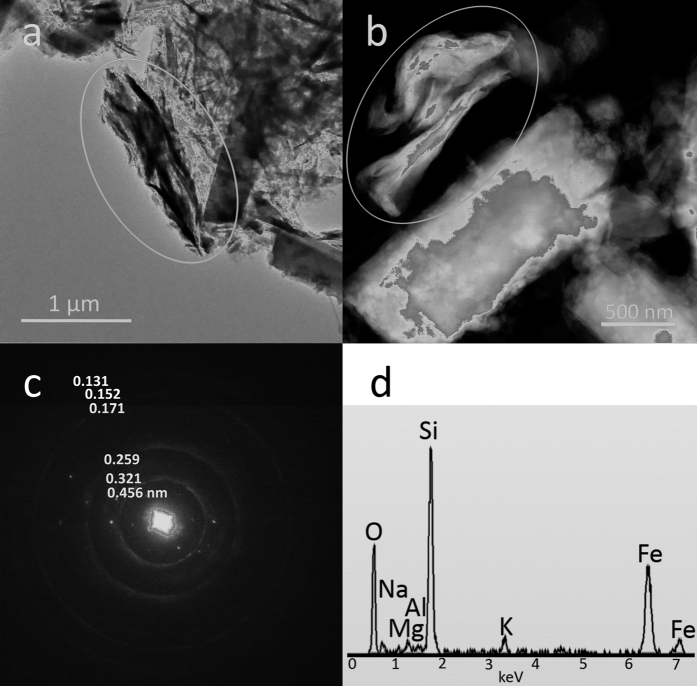
TEM images of nontronite flakes associated to the erionite-Na fibres. TEM images and relative EDS spectra showing the presence of micro- to nano-metric flakes of nontronite close to the erionite fibres. The *d*-spacings of the rings of the electron diffraction pattern are compatible with the major diffraction maxima of nontronite.

**Figure 5:**
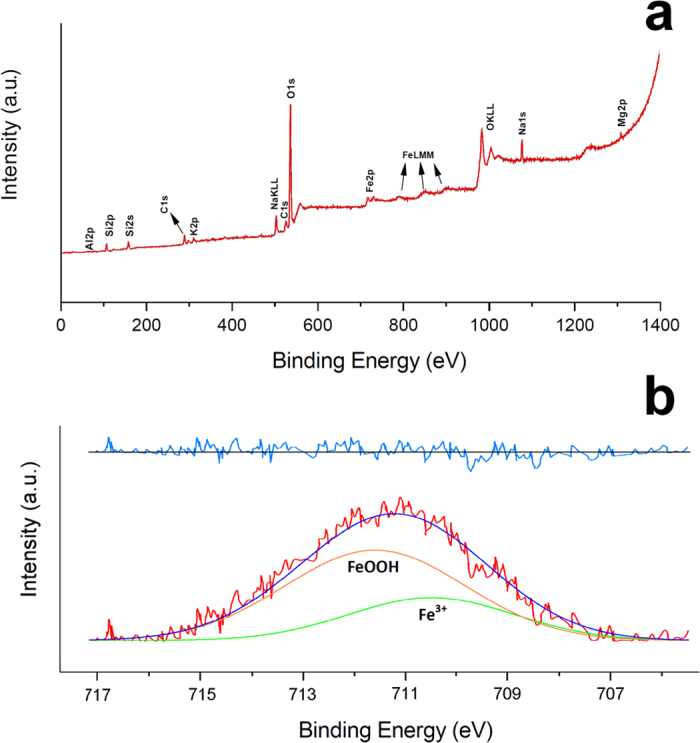
XPS spectra collected on fibrous erionite. The full XPS spectra collected on fibrous erionite (**a**) and the the Fe 2p_3/2_ spectra curve fit (**b**) with the experimental curve (red), the calculated curve (blue), the single contributions (green and orange), and the difference curve (light blue on the top).

Figure 1 portrays an example of high resolution TEM image of the erionite sample with single fibres characterized by stacking disorder along the *c* axis and the relative diffraction pattern from an area of high cristallinity. As already evidenced in the preliminary TEM study^33^, rare iron-rich nanoparticles were originally discovered at the surface of the erionite fibres. Figure 2 displays one of these particles with the relative EDS analysis revealing that such clusters are enriched in iron with respect to the bulk. Other nanoparticles of smaller diameter (around 10 nm) were also observed in the same sample during the TEM sessions. Figure 3 are FEG/SEM images and relative EDS analysis on the erionite sample revealing the presence of micrometric particles at the surface of the erionite fibres. From the EDS analysis on the brighter clusters, it is clear that they are enriched in iron with respect to the fibre bulk. Figure 4 is another beautiful TEM picture witnessing the presence of micro- to nano-metric flakes associated to the erionite fibres. The size of each single particle is certainly nanometric (Fig. 4a,b). The analysis of the electron diffraction rings produced by such aggregates (Fig. 4c) permitted to identify the impurity as nontronite, with the rings at a *d*-spacing of 0.454 (*020*), 0.321 (*003*), 0.259 (*200* and 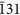), 0.171 (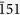), 0.152 (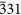), 0.131 (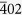. 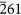. *027*, and *136*) nm. The first ring corresponding to the *001* is hidden by the direct beam. Once again, these flakes are enriched in iron with respect to the bulk of the fibres (Fig. 4d). The EDS spectra also revealed a concentration of Mg in correspondence with the clay flakes. Figure 5a is the full XPS spectrum of the erionite sample, giving indication on the chemical nature of surface iron in erionite. To identify the fractions of different oxidation states of iron, the peak deconvolution of the region with the Fe 2p_3/2_ has been made (Fig. 5b) following the same procedure used in Fantauzzi *et al*.^34^. The peak fit (R^2^ = 0.99909) permitted to identify and quantify both the Fe^3+^ (30%) and Fe^3+^OOH (70%) contributions.

## Discussion

### The nature of iron in fibrous erionite

The XAFS and Mössbauer spectroscopy of fibrous erionite-Na from Jersey, discussed in detail in Pollastri *et al*.^33^, give evidence of the exclusive presence of Fe^3+^ hosted in an octahedral (6-fold) environment. In particular, the EXAFS data identify a first shell of six oxygen atoms at a mean distance of 0.2011 nm, very close to the theoretical distance Fe^3+^-O (0.2015 nm) for octahedral iron^33^. The Mössbauer data also show the absence of magnetic sextets typical of iron oxide, indicating that the iron-bearing phase must have a dimension under the resolution limit of the measurement at RT, namely <18 nm^35^.

The outcome of the structure refinement confirms the absence of structural iron in natural fibrous erionite ruling out both Fe^2+^/Fe^3+^ in place of Si^4+^/Al^3+^ in the zeolite framework and Fe^3+^/Fe^2+^ in the extraframework cavities (micropores) as octahedral Fe(H_2_O)_n_ clusters.

**Figure 6:**
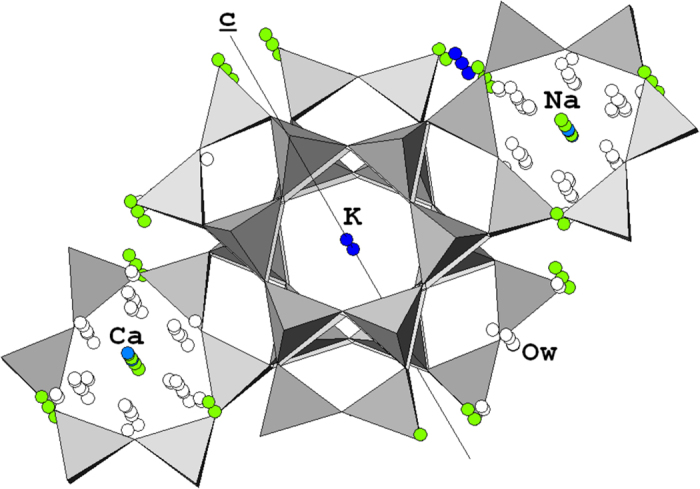
A plot of the calculated structure of fibrous erionite from Jersey (Nevada, USA). A plot of the calculated structure of fibrous erionite from Jersey (Nevada, USA) in the *a*-*b* plane with the position of the extraframework cations. Legend: green balls = Na; light blue balls = Ca; cobalt blue balls = K; white balls =  H_2_O molecules.

Concerning the framework, the two independent tetrahedral sites T1 and T2 (building respectively, the D6R and the S6R) are characterized by disordered Si/Al distribution. In fact, the <T1-O> − <T2-O> is −0.0044 nm, indicating the preferential partition of Al for T2, in agreement with the literature data^8^,^9^,^10^. As far as the extraframework cations are concerned, K atoms are found at the centre of the cancrinite cage (Fig. 6)^9^,^10^,^36^. K is bonded to 12 framework oxygen atoms (6 O2 and 6 O3) at a mean bond distance of 0.317 nm, and the K site is fully occupied (see Table 1), in agreement with Ballirano *et al*.^10^. A minor fraction of K ions are found in the erionite cage, in correspondence with the crystallographic site labelled Ca4 (K2 in Ballirano *et al*.^10^). The coordination of K in that site is also 12-fold with 6 framework oxygen atoms (4 O1 and 2 O4) and 6 H_2_O molecules (4 O_w8_ and 2 O_w12c_) at a mean bond distance of 0.3192 nm. The erionite cage hosts a number of Na and Ca ions in a disordered fashion (Fig. 6). Site Ca1 which hosts minor Ca ions is close to the centre of the single 6-membered ring shared by two adjacent erionite cages, forming the base of the cavity. Ca ions are 12-fold coordinated by H_2_O molecules only (O_w8_, O_w10_, O_w12b_, and O_w12c_) at a mean bond distance of 0.244 nm. Although Ca1 is invariably coordinated to H_2_O molecules only, a different coordination number is reported for erionite-Ca^9^ (3) and erionite-K^10^ (11 if distances <0.3 nm considered). Na ions are distributed over three distinct sites (Ca2, Ca3, and Ca4b). Ca2 is close to the centre of the upper half of the cavity where Na is in 6-fold coordination with H_2_O molecules (3 O_w8_ and 3 O_w12b_) at a mean bond distance of 0.223 nm. The same coordination number was found for erionite-K^10^ (6) whereas a different number is reported for erionite-Ca^9^ (6–12). The latter also includes a connection with the framework oxygen atom O5 at 0.2922 nm. Ca3 is shifted along the 3-fold axis in the erionite cage, close to the 6-membered ring. Na in Ca3 site has a 6-fold coordination with H_2_O molecules (3 O_w9_ and 3 O_w12c_) at a mean bond distance of 0.234 nm. The coordination number of Ca3 is larger for erionite-Ca (9–12)^8^,^9^. Na is also hosted in the new site Ca4b, shifted with respect to the main 3-fold axis with Na that again displays a 6-fold coordination with H_2_O molecules (2 O_w8_, 2 O_w11_, O_w12b_ and O_w12c_) at a mean bond distance of 0.2757 nm. Site Ca3 and Ca4b are not reported for erionite-K^10^.

Attempts were made with the aim to locate Mg ions in the structure but they all invariably failed, indicating that minor Mg from the chemical analysis belongs to impurities of clay phases (see below) other than to the erionite structure.

The experimental evidences in our hands confirm that the investigated erionite fibres from Jersey do not contain structural Fe^3+^ and point to Fe^3+^ associated to iron-rich impurities, corroborating model (3) (see Introduction).

The fact that iron is not found in the erionite structure of natural samples has also a sound petro-genetic basis. Erionite is a sedimentary zeolite likely formed in open hydrologic systems or hydrothermal environment during the so-called *zeolitization* process^37^,^38^. During the process, iron eventually present as Fe^2+^ in the host tuffs, is leached, oxidized and precipitated later as secondary iron-bearing phases like iron hydroxides. Even the hydrothermal synthesis in laboratory aimed at reproducing the natural system evidenced that the majority of Fe^3+^ ions of the zeolitized samples is not included in the zeolitic structures as isomorphous substituent or exchanged cation, but belongs to ancillary phases^39^.

The fibrous erionite sample is kind of heterogeneous as it contains iron-bearing impurities of different nature. We have found amorphous iron-oxide/hydroxide nanoparticles with diameter around 10 nm or greater (around 50 nm: Fig. 2). Such nanoparticles resemble those observed by Ballirano *et al*.^10^ in erionite-K from Rome (Oregon, USA) where most of iron was attributed to Fe^3+^-bearing, super-paramagnetic, oxide-like nanoparticles of size between 1 and 9 nm. The FEG/SEM study also revealed the presence of micrometric particles, possibly aggregates of nano/micro-particles of iron oxides/hydroxides, at the surface of the erionite fibres (Fig. 3). The same micro-aggregates have been reported for erionite-K from Rome^10^,^30^, and for erionite-K from Karlik (Cappadocia, Turkey)^30^. According to Croce *et al*.^30^, erionite-K from Rome contains iron-bearing microcrystals of hematite (α-Fe_2_O_3_), goethite (α-FeOOH), and jarosite KFe_3_(SO_4_)_2_(OH)_6_ at the surface of the fibres, whereas the erionite-K from Karlik shows iron-bearing sub-micrometric crystals of hematite and goethite at the surface of the fibres. High resolution TEM imaging also revealed the presence of micro- to nano-metric flakes enriched in iron with respect to the bulk of the fibres (Fig. 4). Such flakes have been identified as nontronite. Nontronite also explains the minor amount of Mg detected by the chemical analysis of the sample. The presence of nontronite as clay impurity in fibrous erionite samples has been already reported^29^. Hence, with the results of our study we have verified that the very same erionite sample may contain different iron-bearing impurities, explaining why different authors see impurities of different kind in natural fibrous erionite.

The coordination environments of iron of the impurities is also compatible with the results from the XAFS and Mössbauer study^33^. Goethite, hematite and nontronite all have Fe^3+^ in octahedral environment. Although uncommon, nontronite may contain very minor tetrahedral Fe^3+^ if the Fe_2_O_3_ content is >37%^40^. The mean calculated Fe^3+^-O bond distance in goethite^41^, hematite^42^ and nontronite^43^ are 0.2013, 0.2029, and 0.2013 nm, respectively. Such values are in agreement with the EXAFS data showing a first shell of six oxygen atoms at a mean distance of 0.2011 nm.

### Is the toxicity of fibrous erionite related to iron?

Fibrous erionite is considered more potent than chrysotile in causing MM^44^ and the understanding of toxicity mechanisms of this peculiar mineral fibre is still an open issue. Many authors believe that iron plays a key role in determining the toxicity of this fibre as erionite toxicity has been partly ascribed to ion-exchanged and/or surface-deposited Fe participating in Fenton chemistry^10^,^45^,^46^. Along the same research line, it was found that erionite from Rome, Oregon and Pine Valley, NV (adjacent to Jersey, NV) show quite different effects in animal studies. The sample from Rome is iron-rich whereas the sample from Pine Valley has very low iron content. The results indeed show that Rome erionite with Fe in some form, is more potent than Fe-poor erionite^47^. This result has important consequences for other animal studies because most laboratory experiments have used Rome erionite and may be the main basis for the general conclusion that erionite is more carcinogenic than asbestos minerals.

The results of our study clearly demonstrate that iron is not part of the erionite crystal structure but is associated to impurities, mostly concentrated at the surface of the fibres. If iron is not part of the structure, its role should be carefully reconsidered and a specific model of bioavailability should be developed to understand the carcinogenicity of erionite fibres. In fact, there is a sharp distinction between the iron content of a fibre and the iron content of a particle like an iron oxide, present as impurity. Toxicity due to release of HO^•^ is related to the content of active surface structural iron of a fibre as the latter may prompt the activation and release of H_2_O_2_ (or radical species O_2_^−^ or free oxygen) during macrophage frustrated phagocytosis. The proof that structural iron of a fibre is a key factor is found in Gazzano *et al*.^48^ who reported that synthetic stoichiometric chrysotile nanofibers, devoid of iron did not exert genotoxic and cytotoxic effects. On the contrary, the same nanofibers, loaded with 0.57 wt% and 0.94 wt% iron, induced DNA strand breaks, lipoperoxidation, inhibition of redox metabolism and alterations of cell integrity, similarly to natural chrysotile. On the other hand, the iron in a sub-spherical particle or nanoparticle will not be active (and hence toxic) as it is successfully phagocytized by macrophages, with no H_2_O_2_-mediated release of HO^•^. If we share this view, iron should not be involved in the toxicity mechanism of erionite fibres as the low iron content is actually *alien* Fe^3+^, in the form of Fe^3+^-rich oxide nanoparticles. If *alien* iron in fibrous erionite is irrelevant for its potential toxicity, other factors should be invoked. One of these is certainly the high biopersistence of this fibre species in both extracellular and intracellular environment. Another factor that has been recently disclosed is that fibrous erionite may contain appreciable amounts of toxic elements such as As, Be and Pb. Since there is convincing evidence of a relationship between lung cancer mortality and cumulative As, Be, and Pb exposure, the toxicity of this fibre species may also be related to the synergetic effect of these contaminants^49^.

**Figure 7:**
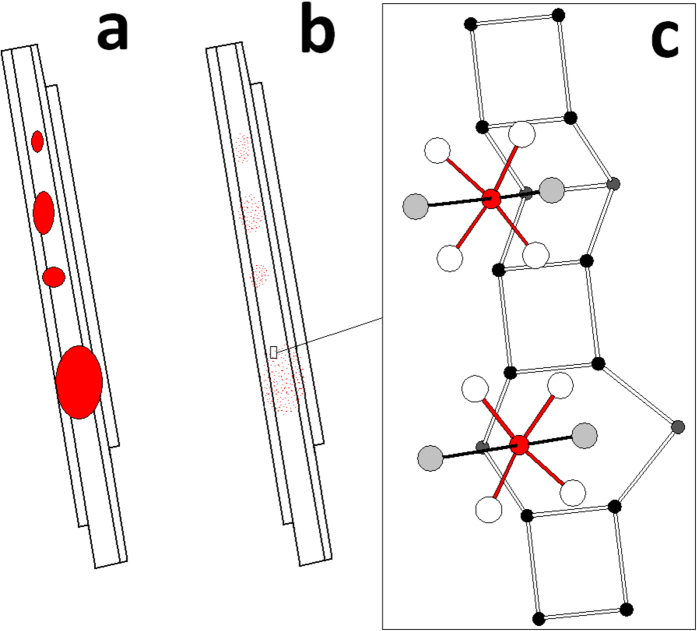
Location of possible active iron sites at the surface of erionite fibres. If the iron-rich nanoparticles at the surface of the erionite fibres (**a**) are dissolved during alveolar macrophage phagocytosis at pH = 4–4.5, the residue of the dissolution at the surface should be iron atoms at specific surface sites (**b**) anchored to the windows of the zeolite channels. One of these sites may be found at the windows of the 6-membered rings anchored to 4 oxygen atoms of the framework and 2 water molecules to form a distorted octahedral coordination (**c**).

There is an alternative perspective to the proposed model of *alien* iron in fibrous erionite: although micro-particles of iron oxides (namely goethite and hematite) are stable in acidic environment^50^,^51^, iron rich nano-particles and especially nontronite are not^29^. Hence, the latter can be dissolved during alveolar macrophage phagocytosis when the fibres are engulfed in the lysosome sacks with acidic environment (pH = 4–4.5) (Fig. 7a). The dissolution of such phases present at the surface of the erionite fibres should leave a residue of iron atoms at specific sites anchored to the surface windows of the zeolite channels (Fig. 7b). One of these sites may be at the windows of the 6-membered rings anchored to 4 oxygen atoms of the framework (specifically O2 and O3) and 2 H_2_O molecules to gain an octahedral coordination (Fig. 7c). With reference to the erionite unit cell, the position of that iron site should be x/*a* = 0.1116; y/*b* = 0.0; z/*c* = 3/4. The surface sites for iron may become active as low nuclearity groups. Although the site described here hosts Fe^3+^, active sites have already been reported for erionite as isolated (FeO)^2+^ structures as preferred candidate active sites [(H_2_O)_5_FeO]^2+^ due to their low nuclearity^52^. Instead, paired Fe^2+^ − Fe^2+^ active species or Fe_x_O_y_ clusters eventually entrapped into the zeolite framework cavities are considered less active.

A final speculative remark concerns the possibility that in open environment erionite fibres undergo reduction of surface iron by microbial species. This is a common biochemical process observed for many iron-bearing minerals^53^. The production of surface Fe^2+^ enhances the toxicity of the fibres as it directly promotes the formation of reactive HO^•^ species by the well known Fenton reaction sequence.

## Methods

### Sample selection and characterization

A natural fibrous erionite-Na from Jersey, Nevada (USA) has been selected for this study. The quantitative chemical composition was determined by Electron Micro Probe Analysis (EMPA) using a JEOL 8200 Super Probe instrument with W hairpin type filament and minimum accelerating voltage of 30 kV. For the determination of the water content, thermogravimetric and differential scanning calorimetry (TG/DSC) analysis were performed using a Netzsch STA 449 C Jupiter and published elsewhere^49^. The final crystal chemical formula was calculated, after renormalization of the chemical analysis with a H_2_O content of 18.5 wt%, which corresponds to about 30 atoms per formula unit (*apfu*), on the basis of 36 (Si + Al) *apfu*.

The calculated formula is: Fe^3+^_0.29_(Na_5.35_K_2.19_Ca_0.15_Mg_0.11_Ti_0.05_)_7.85_[Si_28.01_Al_7.90_]_35.91_O_72_·28.13H_2_O. Iron has been intentionally excluded from both the group of framework and extraframework cations because of its uncertain location. The specimen contains less than 1 wt% of clinoptilolite. Other impurities are below the detection limit of X-ray diffraction. The detailed electron microscopy study of the fibres was performed using a FEG/SEM FEI Quanta Nova NanoSEM 450 instrument, a JEM 2010 JEOL TEM a and a FEI Tecnai STEM. As anticipated, the chemical environment of iron was previously determined using Mössbauer and X-ray absorption spectroscopy (XANES and EXAFS)^33^. XPS spectra were acquired by a V.G. ESCALAB MKII using a Mg anode (Kα radiation line at 1253.6 eV unmonochromatised) as X-ray source, operating at a voltage of 10 kV and a power of 240 W. Spectra were acquired at pass energy of 20 eV and step channel of 0.1 eV.

### Synchrotron powder diffraction and Rietveld refinement strategy

Synchrotron X-ray powder diffraction (XRPD) patterns were collected at the MCX beamline at the synchrotron facility of ELETTRA (Trieste, Italy). Data were collected with an analyzer crystal detector in Debye-Scherrer mode, at two different energies: close to the absorption K-edge of iron (≈7 keV, λ 0.17428 nm) and far away from the absorption edge of iron (≈10 keV, λ 0.12408 nm). In this way, potential anomalous dispersion effects due to iron could be evaluated because at the absorption energy anomalous intensities from the diffracting planes of the crystalline phases (both erionite or impurities) containing iron arise. Such effect can be modelled within the Rietveld refinement and permits to eventually locate iron in the crystal structure. Structure refinements were accomplished using the GSAS package^54^ and its graphical interface EXPGUI^55^. The starting structure model (space group *P*6_3_/*mmc*) was taken from Gualtieri *et al*.^9^. The structure factors were calculated using the formal scattering factors for neutral atoms. The background profile, due to the scattering of the capillary, was successfully fitted with a Chebyshev polynomial function with 28 coefficients. The diffraction peak profiles were modelled using a pseudo-Voigt function with a θ-independent Gaussian and two Lorentzian coefficients. The unit-cell parameters and phase fraction were refined together with the atomic coordinates, the atomic site occupancies for extraframework positions, and the isotropic atomic displacement parameters. Soft constraints on tetrahedral bond lengths were imposed and used as additional observations in the earlier stages of the refinement procedure. The weight of the constraints was progressively reduced to zero in the later stages. Difference-Fourier maps of the electron density function were calculated from the refined model and were useful for the location of residual electron density, corresponding to extraframework cations or H_2_O molecules. The structure refinement using the data collected at the iron absorption edge were conducted in the attempt to locate structural iron in the zeolite structure. The population of the crystallographic sites which were supposed to host iron was refined using anomalous scattering factors f′ (−7.98) and f″ (1.862) for Fe^3+^ at 7112 eV.

As an example, the graphical output of the Rietveld refinement using the data collected at the iron absorption edge is shown in Supplementary Fig. 1. Miscellaneous statistics of the refinements and calculated structure parameters are reported in Supplementary Table 1. Full structural data of erionite has been deposited under the form of CIF file.

## Additional Information

**How to cite this article**: Gualtieri, A. F. *et al*. Where is iron in erionite? A multidisciplinary study on fibrous erionite-Na from Jersey (Nevada, USA). *Sci. Rep.* **6**, 37981; doi: 10.1038/srep37981 (2016).

**Publisher's note:** Springer Nature remains neutral with regard to jurisdictional claims in published maps and institutional affiliations.

## Supplementary Material

Supplementary Information

## Figures and Tables

**Table 1:** Calculated distances and angles of fibrous erionite from Jersey (Nevada, USA).

Framework
T1-O1 0.1613(1) nm
T1-O2 0.1612(1) nm
T1-O3 0.1617(1) nm
T1-O4 0.1623(1) nm
mean 0.1616 nm
O1-T1-O2 111.03(8)°
O1-T1-O3 108.54(7)°
O1-T1-O4 106.94(6)°
O2-T1-O3 108.67(8)°
O2-T1-O4 110.69(7)°
O3-T1-O4 110.95(8)°
mean 109.47°
T2-O1 0.1675(1) nm
T2-O1 0.1675(1) nm
T2-O5 0.1641(1) nm
T2-O6 0.1651(1) nm
mean 0.1660
O1-T2-O1 108.52(7)°
O1-T2-O5 114.50(7)°
O1-T2-O6 105.92(8)°
O1-T2-O5 114.50(4)°
O1-T2-O6 105.92(6)°
O5-T2-O6 106.76(7)°
mean 109.35°
**Extraframework**
Ca1 = atom type Ca
Ca1-Ow8 0.227(1) nm × 3
Ca1-Ow10 0.210(1) nm × 3
Ca1-Ow12b 0.258(1) nm × 3
Ca1-Ow12c 0.282(1) nm × 3
mean 0.244 nm
Ca1-Ow9 0.185090(1) nm × 3
Ca1-Ow11 0.164082(1) nm × 3
Ca2 = atom type Na
Ca2-Ow8 0.227(2) nm × 3
Ca2-Ow12b 0.219(1) nm × 3
mean 0.223 nm
Ca3 = atom type Na
Ca3-Ow9 0.207(2) nm × 3
Ca3-Ow12c 0.2604(9) nm × 3
mean 0.234 nm
Ca3-Ow7 0.275(1) nm × 3
Ca3-Ow8 0.288(1) nm × 3
Ca3-Ow10 0.277(1) nm × 3
Ca3-Ca1 0.081(1) nm
Ca3-Ow10 0.163(2) nm × 3
Ca3-Ow11 0.179(2) nm × 3
Ca4 = atom type K
Ca4-O1 0.326(1) nm × 4
Ca4-O4 0.312(2) nm × 2
Ca4-w8 0.332(2) nm × 4
Ca4-w12c 0.287(1) nm × 2
mean 0.3192 nm
Ca4-w9 0.2379(9) nm × 2
Ca4-w12b 0.204(2) nm × 2
Ca4b = atom type Na
Ca4b-Ow8 0.2598(9) nm × 2
Ca4b-Ow11 0.2979(9) nm × 2
Ca4b-Ow12b 0.285(2) nm
Ca4b-Ow12c 0.254(1) nm
Mean 0.2757 nm
Ca4b-Ca4b 0.167(9 nm
Ca4b-K2 0.084(1) nm
Ca4b-Ow9 0.169(1) nm
Ca4b-Ow12b 0.125(1) nm
K = atom type K
K-O2 0.299(1) nm × 6
K-O3 0.335(2) nm × 6
mean 0.317 nm
**Water molecules**
Ow8-Ow11 0.163(1) nm
Ow8-Ow12b 0.178(1) nm
Ow8-Ow12b 0.178(1) nm
Ow9-Ow10 0.165(3) nm
Ow9-Ow11 0.176(1) nm × 2
Ow9-Ow12b 0.171(1) nm
Ow9-Ow12c 0.146(2) nm
Ow10-Ow10 0.167(1) nm
Ow10-Ow11 0.209(2) nm × 2
Ow10-Ow12c 0.115(1) nm
