# Supplementary material for: Where is iron in erionite? A multidisciplinary study on fibrous erionite-Na from Jersey (Nevada, USA)
Source: Sci Rep. 2016 Nov 28;6:37981. doi: 10.1038/srep37981 (PMC5125093; doi:10.1038/srep37981)
Supplement: Supplementary Information [file srep37981-s1.pdf]

SUPPLEMENTARY INFORMATION

# Where is iron in erionite? A multidisciplinary study on fibrous erionite-Na from Jersey (Nevada, USA)

**Alessandro F. Gualtieri[1], Nicola Bursi Gandolfi[1], Simone Pollastri[1], Kilian Pollok[2], Falko Langenhorst[2]**

[1]Chemistry and Earth Sciences Department, The University of Modena and Reggio Emilia, Via Campi 103, I-41125 Modena (Italy). [2]Institut für Geowissenschaften Mineralogie, Friedrich-Schiller-Universität Jena, Carl-Zeiss-Promenade 10, D-07745 Jena (Germany). Correspondence and requests for materials should be addressed to A.F.G. (email: alessandro.gualtieri@unimore.it)

**Supplementary Figure 1. The graphical output of the Rietveld refinement of fibrous erionite.** The graphical output of the Rietveld refinement of fibrous erionite with the observed vs. calculated plots, curve of the differences and reflection markers of both erionite and clinoptilolite.

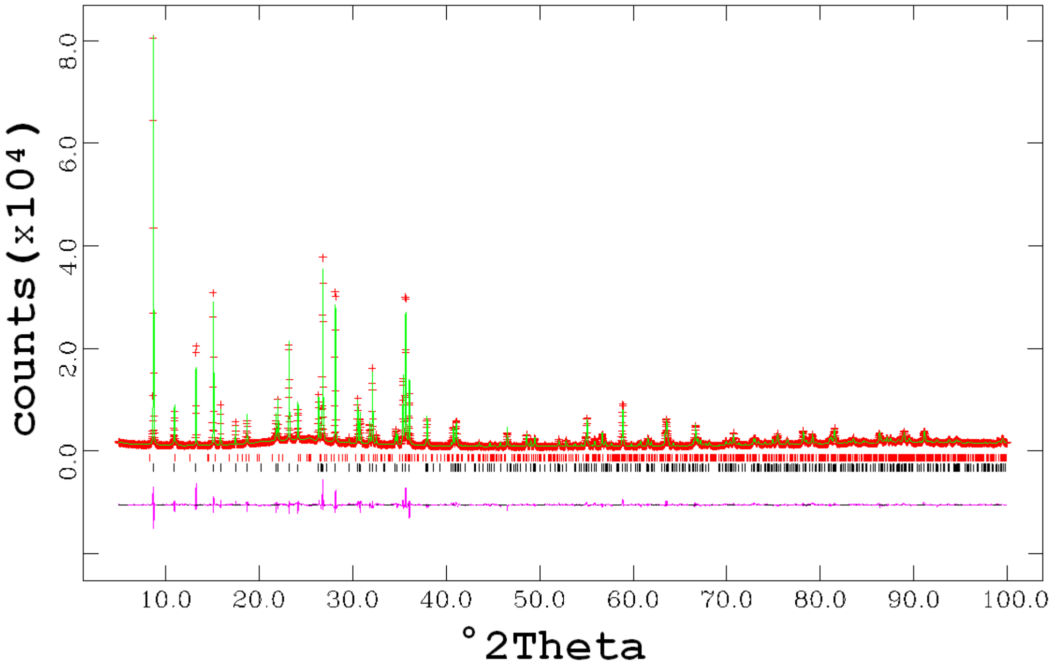

**Supplementary Table 1. Rietveld refinement statistics and structural parameters of fibrous erionite from Jersey (Nevada, USA)**.

| Rietveld agreement factors | | | | | | |
|---|---|---|---|---|---|---|
| $R_{wp}$=7.26% | $R_p$=5.28% | $\chi^2$=9.86 | $R(F^2)$=6.45% | $D_{wd}$=0.339 | $N_{obs}$=9510 | $N_{ref}$=1424 |
| Refined unit cell in space group $P6_3/m\ m\ c$ | | | | | | |
| a=1.3238496(72) nm | | c=1.5084412(94) nm | | $V_{cell}$=22.89477(27) $nm^3$ | | |
| site | x/a | y/b | z/c | Site multiplicity | Occupancy (atom type) | Uiso ($Å^2$) |
| T1 | 0.0003(2) | 0.2334(2) | 0.1041(1) | 24 | 1 | 0.005(2) |
| T2 | 0.0935(2) | 0.4225(3) | ¼ | 12 | 1 | 0.005(2) |
| O1 | 0.0220(3) | 0.3464(2) | 0.1599(3) | 24 | 1 | 0.026(3) |
| O2 | 0.1007(2) | 2x(4) | 0.1239(3) | 12 | 1 | 0.037(3) |
| O3 | 0.1241(2) | 2x(4) | 0.6331(3) | 12 | 1 | 0.018(2) |
| O4 | 0.2645(3) | 0 | 0 | 12 | 1 | 0.007(3) |
| O5 | 0.2337(3) | 2x(6) | ¼ | 6 | 1 | 0.036(4) |
| O6 | 0.0809(6) | 0.540(1) | ¼ | 6 | 1 | 0.030(3) |
| Ca1 | 1/3 | 2x | 0.904(1) | 4 | 0.0375(Ca)* | 0.109(4) |
| Ca2 | 0.3333 | 2x | 0.107(1) | 4 | 0.50(6)(Na) | 0.111(5) |
| Ca3 | 0.3333 | 2x | 0.650(4) | 4 | 0.17(5)(Na) | 0.111(5) |
| Ca4 | ½ | 0 | 0 | 6 | 0.03(K)* | 0.025(4) |
| Ca4b | 0.5364(4) | 0.0728(7) | 0.005(1) | 12 | 0.44(5)(Na) | 0.111(5) |
| K | 0 | 0 | ¼ | 2 | 1(K) | 0.025(4) |
| $O_{w7}$ | 0.2329(6) | 2x(12) | ¾ | 6 | 1 | 0.165(5) |
| $O_{w8}$ | 0.260(1) | 2x(2) | 0.005(1) | 12 | 0.24(3) | 0.165(5) |
| $O_{w9}$ | 0.414(2) | 2x(4) | 0.912(2) | 12 | 0.32(2) | 0.165(5) |
| $O_{w10}$ | 0.398(1) | 2x(2) | 0.695(2) | 12 | 0.31(3) | 0.165(5) |
| $O_{w11}$ | 0.262(1) | 2x(2) | 0.602(1) | 12 | 0.23(4) | 0.165(5) |
| $O_{w12b}$ | 0.413(1) | 2x(2) | 0.025(1) | 12 | 0.41(3) | 0.165(5) |
| $O_{w12c}$ | 0.554(1) | 2x(2) | 0.172(2) | 12 | 0.31(4) | 0.165(5) |

*constrained to the values of the chemical analysis.
